# Chloroform in Surgical Operations

**Published:** 1850-09

**Authors:** John A. Halderman

**Affiliations:** Carlinville, Illinois


### ARTICLE II.

*Report of Surgical Operations under the use of Chloroform.*

By John A. Halderman, M. D., of Carlinville, Illinois.

From the interest manifested by the medical profession and the public in general, relative to the use and properties of anæsthetic agents in medical and surgical practice, I have been induced to communicate to your valuable Journal the result of my experience in the use of chloroform in a few cases that seemed, in my judgment, to indicate its employment. As facts and experience, when carefully and accurately reported, will always subserve the true and practical interest of medical science; and as there is so much diversity of opinion with regard to the influence of this remedy on the animal economy, I have thought the following results of my limited observations and practice might serve, in some degree, to call the attention of some abler members of the profession from among the numerous readers and contributors of scientific medicine.

CASE I.—Mr. A. C. Stephenson, a gentleman of middle age, and of delicate constitution, was attacked, in May last, with erysipelas of the right side of the chest. So malignant was the inflammatory action as to result in extensive suppuration under the fascia and muscles of the part, requiring the matter to be discharged, for which I was called to operate.

From the excessive sensibility of the nervous system, I thought the use of chloroform was clearly indicated, preparatory to the operation. A small incision had been made by Dr. A. Miller, the attending physician, who, with Dr. D. S. Brock, assisted in the case. One drachm of the Chlororoform was inhaled in about five minutes, when complete insensibility was induced. I immediately commenced the operation by making a second incision with a thumb lancet, three inches lower down than the one which had been made previously. I then introduced a grooved director into the lower orifice and gently pushed it through the upper opening. A probe-pointed bistoury was then carried along the groove of the director from one opening to the other, through the inflamed skin and subjacent parts, exposing freely the bottom of the abscess, at least three inches in length. The matter was all broken up with a scoop and discharged. During the whole of the operation there was not the slightest evidence of either pain or consciousness on the part of the patient. A few minutes after the operation was over, he recovered the possession of his mind and feelings, but affirmed that he knew and felt nothing that had been done. He soon got well.

CASE II.—Master N. B. Holly, a boy aged fifteen years, of a dwarfish and sickly growth from his infancy; was brought by his father to my office last September, for the extirpation of a large and suspicious tumor from the inside of the upper eyelid of the left eye. I placed him on his back on the office counter, with his head elevated and fairly exposed to the light of the window, and administered to him a little over half a drachm of chloroform. Complete loss of sensibility was produced within eight or ten minutes. Dr. E. Wright, my pupil at that time, assisted me with the case.

The eyelid being everted, and the tumor, thrust through with a common tenaculum, was entirely removed with a small scalpel, Considerable hemorrhage ensued, which increased the feebleness of his naturally very defective circulation. His consciousness and sensibility did not return for nearly forty minutes after the operation, when the youth was surprised and rejoiced to learn that the tumor was out. He knew and felt nothing of the operation. At the expiration of a week, his father took him home, perfectly cured.

Case III.—Mr. R. H. King, of Madison county, had his finger contused about the root of the nail, so as partially to destroy its connection. Inflammation with the most exquisite pain supervened. Upon examination I found that the loosened nail was acting as an extraneous substance on the tender and sensitive *quick*. I advised the removal of the nail, under the influence of Chloroform. He was placed upon the bed, took a half-ounce of Chloroform, which induced insensibility for about two minntes, when I pulled the nail off, with a pair of dental forceps, without any pain from, or knowledge of the operation. For the space of fifteen minutes after the operation, Mr. K. continued in a state of mental revery, describing in glowing language the beauties and exquisite grandure of an ideal scenery, which he afterwards compared to a vision of Paradise.

Case IV.—Mr. R. Rodes, aged 60 years, came to consult me last September, relative to a large cancerous tumor of the under lip. I advised its removal with the knife. After a few days attention to his general health, the operation was performed in the following manner, on the second day of October last. After placing him on his back on the office counter, he inhaled one ounce of pure Chloroform during the space of one hour, before complete anæsthesia was induced. I then caught the lip between my finger and thumb, pulled it perpendicularly from the jaw, and with a scalpel made a semi-circalar incision from one angle of the mouth to the other, including the entire lip down to the chin. Both the labial branches of the facial, and the sublingual arteries were taken up and secured by ligatures. The whole operation was performed without the slightest pain or even consciousness being experienced. When the old gentleman recovered his senses and found that the operation was over, he was exceedingly gratified. At the expiration of two weeks the wound was well. Seven months have now elapsed, and there is every appearance of a radical cure of the malady.

CASE V.—Mr. T. R. J. English, aged about thirty-five years, consulted me in March last, relative to the treatment of a malignant cancerous affection, involving the lips and the left angle of the mouth. It had been treated by different physicians to no good purpose; was of six or seven years standing, and seemed clearly, in my mind, to demand, as the only alternative, immediate extirpation. Consequently, on the ninth of April, with the assistance of Drs. L. J. Woods and E. Wright, I operated. Four drachms of Chloroform were first administered, and the operation commenced; but there was an evident cringing from the knife, shewing some sensibility to pain, when I withheld the scalpel and gave two drachms more of Chloroform, which induced complete loss of feeling, and then resumed the operation, and in less than two minutes removed all the cancerous growth, including more than half of each lip, and nearly the whole cheek of the left [illegible]e. The hemorrhage was active and free; the two labial arteries were secured with ligatures, and the main facial artery compressed on the inferior maxillary bone, so as effectually to command the bleeding. No pain of any consequence was felt till after the operation.

Mr. English is now (first day of May) nearly well. There is no visible indication of any morbid or cancerous character in the wound, which will in a few days be entirely well.

The discovery of the anæsthetic properties of Chloroform may be regarded as constituting a new era in the science of therapeutics, by conferring upon operative surgery a complete antidote to pain. But many respectable and intelligent members of the profession still oppose its use, by alleging that in very many cases it is unnecessary, and in most instances liable to produce injurious consequences. And some enter their caveat against its employment in mitigating the pains of parturition, because they are *natural*, and are the penalty for transgression and sin. But objections urged upon such principles would weigh with equal force against all the salutary and saving influences of the remedial scheme of Redemption. But if any man is under obligation to mitigate the sufferings of humanity, and thereby imitate the great Friend of sinners, it is the skilful and pious physician.

Chloroform, I am sure, when carefully and judiciously employed, will never be productive of injury, or be in any wise detrimental to the patient. But like all other potent and efficient remedies, requires to be administered by a skilful Physician—one who understands the physiology of the human system, the probable resources of vital energy, the pathology of the disease, and the laws of life.

